# Employment and working conditions of nurses: where and how health inequalities have increased during the COVID-19 pandemic?

**DOI:** 10.1186/s12960-021-00651-7

**Published:** 2021-09-16

**Authors:** Alba Llop-Gironés, Ana Vračar, Gisela Llop-Gironés, Joan Benach, Livia Angeli-Silva, Lucero Jaimez, Pramila Thapa, Ramesh Bhatta, Santosh Mahindrakar, Sara Bontempo Scavo, Sonia Nar Devi, Susana Barria, Susana Marcos Alonso, Mireia Julià

**Affiliations:** 1 Research Group on Health Inequalities, Environment, and Employment Conditions (GREDS-EMCONET), Department of Political and Social Sciences, Universitat Pompeu Fabra, Barcelona, Spain; 2 Escola Superior d’Infermeria del Mar (ESIMar), Barcelona, Spain; 3 Social Determinants and Health Education Research Group, IMIM (Hospital del Mar Medical Research Institute), Barcelona, Spain; 4 Organization for Workers’ Initiative and Democratization, Zagreb, Croatia; 5 Nurse and Midwife Consultant, London, UK; 6 The Johns Hopkins - UPF Public Policy Center (JHU-UPF PPC), Barcelona, Spain; 7 Transdisciplinary Research Group On Socioecological Transitions (GinTrans2), Universidad Autónoma de Madrid, Madrid, Spain; 8 Universidade Federal da Bahia, Salvador, Brazil; 9 Nurse Consultant, Mexico City, México; 10 Nurse Consultant, Kathmandu, Nepal; 11 Yeti Health Science Academy, Kathmandu, Nepal; 12 Innovative Alliance for Public Health, New Delhi, India; 13 Nurse Consultant, Bologna, Italy; 14 Nurse and Midwife Consultant, Dublin, Ireland; 15 People’s Health Movement, New Delhi, India; 16 Nurse Consultant, Barcelona, Spain

**Keywords:** Nurses, Employment conditions, Working conditions, Policy, Health

## Abstract

**Background:**

Nurses and midwives play a critical role in the provision of care and the optimization of health services resources worldwide, which is particularly relevant during the current COVID-19 pandemic. However, they can only provide quality services if their work environment provides adequate conditions to support them. Today the employment and working conditions of many nurses worldwide are precarious, and the current pandemic has prompted more visibility to the vulnerability to health-damaging factors of nurses’ globally. This desk review explores how employment relations, and employment and working conditions may be negatively affecting the health of nurses in countries such as Brazil, Croatia, India, Ireland, Italy, México, Nepal, Spain, and the United Kingdom.

**Main body:**

Nurses’ health is influenced by the broader social, economic, and political system and the redistribution of power relations that creates new policies regarding the labour market and the welfare state. The vulnerability faced by nurses is heightened by gender inequalities, in addition to social class, ethnicity/race (and caste), age and migrant status, that are inequality axes that explain why nurses’ workers, and often their families, are exposed to multiple risks and/or poorer health. Before the COVID-19 pandemic, informalization of nurses’ employment and working conditions were unfair and harmed their health. During COVID-19 pandemic, there is evidence that the employment and working conditions of nurses are associated to poor physical and mental health.

**Conclusion:**

The protection of nurses’ health is paramount. International and national enforceable standards are needed, along with economic and health policies designed to substantially improve employment and working conditions for nurses and work–life balance. More knowledge is needed to understand the pathways and mechanisms on how precariousness might affect nurses’ health and monitor the progress towards nurses’ health equity.

## Introduction

Nurses and midwives play a critical role in the provision and quality of care and the optimization of health services resources worldwide. This responsibility has turned to be particularly relevant during the current COVID-19 pandemic, where nurses in the public and private sectors are leading COVID-19 care, testing, triage, and management [1], placing them to a unique position able to deal with vaccination and near-future health challenges. With all, nurses had been one of the most affected collectives by the COVID-19. According to the International Council of Nurses (ICN), millions of nurses have been infected with coronavirus since the start of the pandemic. Cumulative number of reported COVID-19 deaths in nurses in 59 countries was 2262 at the end of 2020 [2]. However, this number is likely to be underestimated as the actual number of fatalities of health workers is unknown due to the absence of a comprehensive systematic surveillance system. Although great efforts have been made in recent years to build up an international health information system with a key set of indicators focused on achieving an adequate size and skill mix of nursing personnel to attain various population health goals [3], the last ICN press release reports that standardized and systematic collection of data on infectious and deaths of health workers is not yet happening [4].

Available data show that prevalence of COVID-19 infections and deaths varied by country and regions. For example, the Americas region accounted for more than 60% of the nurse deaths due to the COVID-19, where Brazil, the USA and Mexico have the highest number of deaths [2]. Compared to other health workers, professional nurses and nurse aides are disproportionately affected by COVID-19. CDC data, from six states of the US, show that among the total number of SARS-CoV-2 infection, 32.1% were nurse aides and 29.5% were professional nurses, compared to 3.2% of physicians [5]. Amongst COVID-19-associated hospitalizations in 13 States of the US, professional nurses account for the largest group (35%), followed by nurse aides (15%) compared to physicians (5%) [6]. These inequalities on COVID-19 infection and deaths by country are due in part to a lack or shortage of personal protective equipment (PPE), where is higher in low-income and middle-income countries (LMICs) [7, 8].

Under usual employment and working conditions, health care workers are known to be at risk for depression, stress, anxiety, burnout or insomnia [9]. COVID-19 pandemic has the potential to significantly impact on mental health of health care workers. A systematic review found a prevalence rate of 23.2% of anxiety, 22.8% of depression and 38.9% of insomnia. Moreover, this systematic review also found gender and occupational differences, where female and nurses had higher rates of affective symptoms [10].

Nurses and other health care workers can only provide quality services if their work environment provides adequate conditions to support them [11]. Today, employment and working conditions of many nurses worldwide are precarious [12], and the current COVID-19 pandemic has prompted more visibility to the vulnerability to health-damaging factors of nurses’ globally. Based on the general framework of employment, work and health of Julià adapted from Benach and Muntaner [13–15], nurses’ health is influenced by several factors. This conceptual framework shows how labour market and welfare state policies influenced by power relations impact on employment conditions. Thus, labour regulations influence how employment conditions are regulated through the different types of contract. Labour relations are the ones that influence the process of precarious employment conditions. Precarious employment measured through different dimensions is present to a greater or lesser extent in the different employment conditions that, directly or through working conditions or material deprivation, produce an impact on mental health, the self-perceived health and health inequalities. Furthermore, unpaid household and care work also has an influence on health and health inequalities. As for social and family networks, they also have an impact on health, depending on whether they are present or not. Inequality axes like gender, age, social class, ethnicity/race and migrant status are key relational mechanisms of generating inequalities.

The aim of this review is to explore how structural social determinants may be negatively affecting the employment and working conditions, practice, and nurses’ health, also during the COVID-19 pandemic, at the global level.

## Methodology

A multidisciplinary team consisting of bedside nurses, activists, union members, and researchers, including nurses’ researchers across nine countries in Asia, Europe and Latin America adopted a collaborative process of critical reflections to guide the understanding of the mechanisms by which nurses’ health is affected, pre-pandemic and during current COVID-19 pandemic.

A desk review was conducted on scientific literature and grey literature, including media, reports, and other relevant resources from various countries including Brazil, Croatia, India, Ireland, Italy, México, Nepal, Spain, and the United Kingdom. We also reported about United States, Australia, and conflict zones such as Palestine, as literature was available. Additional information was sought from Ministries of Health.

**Fig. 1:**
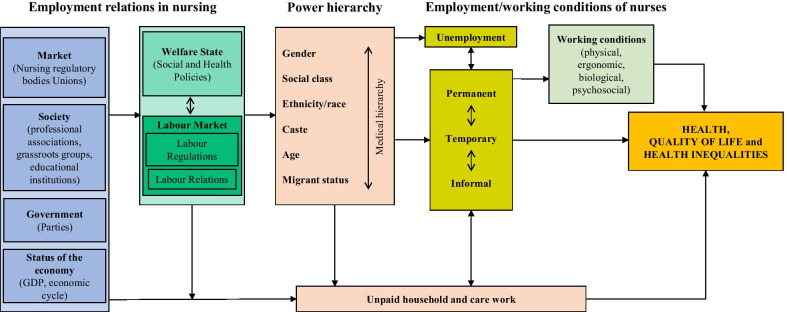
Adapted theoretical framework of the employment, work and the impact on nurses’ health

Pubmed and google searches were conducted combining the key related terms “nurse”, “employment”, “working conditions”, “health” and “COVID-19” according to the guiding priority areas based on the following topics of the theoretical framework [13–15]: employment relations in nursing, power hierarchy, and employment and working conditions of nurses. Figure 1 provides a visual description of the adapted theoretical framework of the employment, work and the impact on nurses’ health.

A total of 77 articles were reviewed, which included 39 research articles, an additional 12 reports, 1 book, 1 thesis, 2 clinical guidance, 2 national official gazette and 20 news articles, as identified by the research team. We based reviews on inputs from the multidisciplinary working group that guided the conceptualization and analysis of the information. Thus, the search was not intended to be exhaustive. We met virtually and discussed by email the emergent findings. Recognizing the importance of the lived experiences, bedside nurses provided a unique contribution to the topic.

## Results

### Employment and working conditions of nurses

#### Employment and working conditions before and during the COVID-19 pandemic

Before the COVID-19 pandemic, there has been a marked increase in precarization and/or informalization of nurses’ employment globally that had an impact on working hours and conditions, minimum wage, social protection, and job insecurity. For example, in Mexico during the period 2005–2018 they reported an increase of informalization among the group of nurses analysed in relation to: (a) the percentage of people without a written contract; (b) the percentage of people with incomes lower than two times the minimum wage; (c) the percentage of nurses without social security, and (d) the percentage of nurses without social benefits [16]. In Catalonia, results of a study show that the highest level of job insecurity occurs among nurse aides and in privately managed nursing homes [17]. In the growing private sector in India where permanent contracts are rare, nurses were often paid less or just above the minimum wage [18]. Similar to the case of Australia, where the employment conditions are worse in the private sector which lead to increased mortality in nursing homes [19]. Also, nurses were already facing structural challenges in many countries as lack of energy and water supply, internet access, enough clean uniforms, appropriate space for breaks, lockers or changing rooms and laundry services, as well as lack of safety, for example during night shifts in isolated areas [12]. Evidence from countries in South Africa [20], Kenya, Tanzania and Uganda [21], Catalonia [17, 22], Brazil [23], Chile [24], Colombia [25], Mexico [26–28], and Thailand [29] show that employment and working conditions of nurses before this pandemic were already associated to poor physical and mental health, and in some cases leading to fatality outcomes as suicide [30, 31]. A particular mention to the nurses working in conflict and war zones. For example, previous to the COVID-19 pandemic, nurses in Palestine reported continuous exposure to traumatic events, and a related feeling of general powerlessness [32]. As the pandemic spread, these occurrences interlinked with additional problems and continuous blockades, including lack of PPE, guidelines and long working hours [33].

During COVID-19 pandemic, nurses, as other healthcare workers, have been working longer hours and/or with different shift patterns, and nursing staff has been reassigned from other clinical areas to, for example, ICU [34], current employment and working conditions of nurses are associated to poor physical and mental in the pandemic context [35, 36]. However, differences in employment and working conditions during the pandemic disaggregated by gender, race or the other inequality axes have not yet been studied.

### Technical division of nurses’ work

A number of countries have a majority of the nursing workforce composed of nurse aides. This is the case of Brazil [37], where currently nurse aides account for the biggest number of deaths of COVID-19, more than of professional nurses [38]. Nursing students, despite traditionally not being considered employees, play a significant role in the health care work during their period of clinical practice and there have been reports of students bullied and breach of working hours agreement, including an enlarged schedule during the night time or weekend hours, as well as a lack of recognition of their work [39, 40]. Also, in Denmark there is evidence that immigrant students from Eastern Europe, Iran, Pakistan, Africa or Asia are at significantly higher risk of being bullied in colleges compared to their native counterparts [41]. In addition, during the pandemic, nursing students of the last year with limited experience in the clinical practice have been sent to the hospitals to work with high-risk patients being underpaid and exposed to big risk-hazards without being qualified to handle such clinical situations [42]. In Spain, nurses who were in an advanced nursing practice course were reabsorbed as clinical nurses with similar responsibilities but underpaid.

### Personal protective equipment

Nurses from different countries reported that the national guidelines and hospital protocols were not well-known among all the nurses working in different settings and sometimes conflicting advice existed, which is in line with current literature [35]. Nurses reported to work based on their experience and knowledge and perceived confusion about adequate procedures for dressing and undressing with the PPE. Lack of PPE was commonly reported by many health workers globally, also in rural areas and the private health sector [43]. They had to purchase their own PPE, when available outside the hospital, reuse old PPE and collaborate together to develop alternative tools for protection [44, 45]. Furthermore, some units such as maternity wards or primary health care facilities have been considered “low risk settings” despite the possibility of positive cases, which again influenced the accessibility of adequate PPE. A report from the United States shows that primary care physicians are the largest subset of physician deaths [46], but data on nursing is unavailable. Evidence shows that nurses who do not consider the availability and quality of PPE to be adequate had significantly higher levels of depression, anxiety, and stress [35, 36, 47].

### Testing and denial of access to health care

Mass testing of asymptomatic health workers during COVID-19 pandemic has been discussed based on the idea that it might not be necessary in health facilities with protocols for PPE [48, 49]. However, one study suggests that weekly screening staff might reduce their contribution to transmission by 25–33% on top of other measures, such as the health workers self-isolation if they develop symptoms [50]. This is also reinforced by CDC advice to test asymptomatic health workers without known or suspected exposure to SARS-CoV-2 working in nursing homes [51]. Currently, there are countries that are systematically offering testing to nurses, such as Italy [52].

There has been less discussion on the determination of payment or insurance coverage of testing or in the case of infection or death of the nurse. For example, in Mexico, a number of nurses have been affected by COVID-19 and they have not had access to testing [53]. Also, in Nepal most of the private health facilities and hospitals have not insured nurses working in COVID-19 wards, who are at higher risk of being infected with COVID-19 [43, 54].

### COVID-19 vaccine

Nurses play a key role in the immunization of the population. However, the majority of the countries that have nurses vaccinated are high- or upper-middle-income countries. Frontline workers are considered, globally, a prioritized group but it can be the case that a healthy young person from a high-income country is vaccinated first than a bedside nurse in a low-income country or nurses working in conflict and war zones [55].

### Power hierarchy: inequality axes

Inequality axes such as gender, social class, and ethnicity/race (and caste), in addition to age and migrant status, are key relational mechanisms that explain why nurses’ workers, and often their families, are exposed to multiple risks and/or poorer health [13, 14]. This may raise questions as for example who is more exposed during health care related work? Power relations and the social positioning in health systems have traditionally valued medical doctors over nurses, and medical structures over communities [56, 57]. This is illustrated by the extraordinary financial compensations provided to healthcare workers exposed during the first wave of the COVID-19 pandemic in some European countries where professional nurses and nurse aides received less, or nothing, compared to their fellow medical doctors. For example, in Catalonia (Spain), compared to medical doctors, professional nurses received 200 euros less, and nurse aides 650 euros less, based on supposed “productivity” criteria [58]. Another example is the United Kingdom that directly overlooked nurses explicitly saying: “reflecting the vital contributions public sector workers make to our country, these pay rises cover the armed forces, teachers, police officers, the National Crime Agency, prison officers, doctors and dentists, the judiciary, senior civil servants and senior military personnel” [59]. Also, in India, doctors were given accommodation best-quality hotels near the hospital while nurses stayed in unsanitary dormitories [60]. Finally, nurses from different countries has reported that the distribution of the PPE has been based on the medical hierarchy rather than the needs of the health workers or the community itself [61]. This hierarchy has been replicated with the administration of vaccines in countries as UK, Italy and Spain.

Despite nurses being the most trusted health workers in clinical settings, discrimination, stigma and violence against nurses as potential vectors of infection are on the rise during the COVID-19 pandemic, hampering nurses’ physical and mental health [34]. Health workers in countries like India are being excluded from communities, evicted from their homes and forced to sleep in hospital bathrooms and on floors for fear that they may be carry the coronavirus [62]. In the city of Rimini in Italy, 70 cars of health workers were damaged overnight outside the hospital [63]. In Mexico, cases of physical and verbal assaults on health workers, including nurses, have been documented both inside and outside hospital facilities, as well as while making home visits to assess patients, and on their way home [64].

### Gender

Nursing workers are predominantly women accounting for 89% of nurses, with variations across world regions; for example, in Africa women represent 76% of the nursing workforce and in South-East Asia 89% [12]. However, only 25% of health leadership positions globally are held by women or nurses [65]. Furthermore, although there is no international data on the gender pay gap disaggregated by health workers, there is evidence of a gender pay gap in the health and social work sectors, both in the public and private sector. On average, the gender pay gap amounts to 26% in high-income and 29% in upper-middle-income countries [66]. In the case of the United States, women nurses earned on average only 91% of what men nurses earned [65], and we can assume that such a gap exists in other countries as well. The association between the gender pay gap and “family gap” is also significant [67, 68]. While for men the salary increases with the number of children, each additional child that women have is associated with a drop in the salary [67]. Yet, there is a lack of such information based on the study of the nursing conditions.

A predominantly female nursing staff requires a range of work time arrangements, such as extended work shifts, night work, and on-call scheduling. The inappropriate use of these arrangements has been shown to negatively impact the health of nursing personnel [69]. During COVID-19 pandemic, the burden of nursing workload for women and their “second shift” as key caregivers within their families added additional stress and fear of infecting family or cohabiters [35].

CDC data from six states of the US suggest that professional nurses, nurse aides, and women are disproportionately affected of COVID-19, despite men being at highest risk of case fatality [5]. Furthermore, during the first wave of COVID-19 pandemic, there has been a lack of consensus and clear information regarding risks for pregnant women workers exposed to COVID-19 which resulted in hospitalizations and deaths. For example, about 18% of the pregnant women analysed needed hospitalization in 13 states of the United States, 2 (1%) were admitted to the ICU, and 1 (0.5%) required invasive mechanical ventilation [6], also there are reports of pregnant women deaths [70].

### Ethnicity/race and caste

There are several examples of discrimination as a result of the race, ethnicity and caste. For example, the nursing workforce in countries like the United States is still predominantly White (75%) as a result of privatization of nursing education that creates unequal access to education, and has left many nurses indebted when they finish their studies, putting pressure on them to take available employment regardless its conditions [71]. Also, colonial legacy and the history of Indian nursing are causes of exploitation and discrimination of Indian women nurses [72].

During COVID-19 pandemic, studies conducted in the United States show that Black essential workers are at higher risk of infection and death of COVID-19 compared to their White counterparts. It has also been shown that Black workers were nearly three times more likely than White workers to hold support roles in health care, such as nurse aides or orderlies [73]. Additionally, CDC data from six States of the United States show that American Indian, Asian and Black health workers are at higher risk of case fatality [5].

### Migrant status

Globally, one in eight nurses practises in a country other than the one where they were born or trained [34]. According to OECD data in 2018, the proportion of migrant nurses accounts for 26% of the nursing workforce in New Zealand, 25% in Switzerland, 18% in Australia or 15% in the United Kingdom [74]. During this pandemic, international recruiters have increased their direct advertising to try and recruit scarce healthcare staff from low- and lower-middle income countries in Africa, Asia and the Caribbean [34]. Discrimination and racism at work in terms of lack of job opportunities, poor career progression or a poor learning environment have been identified as the cause of worse health among migrant and minority nurses compared to native-born nurses [75, 76].

In some countries in Europe during COVID-19, there are reports of migrant nurses unable to visit relatives in the country of origin because the hospital administration is not allowing paid or unpaid leave. In addition, many nurses who migrated in search of better job opportunities in the UK were held up and were not able to register due to COVID-19 and the lockdown. The Nursing and Midwifery Council enabled temporary registration for migrant nurses who completed competitive skill examinations, but those who had not finished were forced to wait for more than 2–3 months for such registration, which hampered their right to higher wages. In India, the United Nurses Association arranged safe repatriation of nurses stranded in Saudi Arabia.

Countries are failing to evaluate and respond to the impact of COVID-19 on the physical and mental wellbeing of migrant workers [77] and data on SARS-CoV-2 infection and COVID-19 deaths in migrant nurses is not systematically collected.

### Age

Available information from 106 countries indicates that 17% of the nursing workforce are aged 55 years or above [12]. Regional variations in formally employed workforce are, however, important. For example, in the Eastern Mediterranean Region there are 14 young nurses for every one approaching retirement; in contrast, the Americas this ratio is 1.2:1, and in Europe and Africa it is 1.9:1, indicating a much smaller replacement pool [12].

Exemptions of COVID work for nurses that are more than 50 years of age were not implemented. Many senior nurses were called to work to fill needed care work and retired nurses are working in the COVID vaccination campaigns [78]. CDC data from six states of the United States show that professional nurses and nurse aides are disproportionately affected and the group of age over 55 years has a significant probability of case fatality [5].

### Employment relations in nursing

The macro-structural framework encompasses the broader social, economic, and political system that exerts significant power over the distribution of resources in a society, shedding light on the complex health and health care politics, and how the redistribution of power relations creates new policies regarding the labour market and the welfare state, namely labour standards, occupational health and safety regulations, and union protections, among many other things [13, 14, 79].

### Unions, civil society, and collective bargaining

Nursing professional associations, educational institutions, nursing regulatory bodies and unions, nursing student and youth groups, grassroots groups, and global campaigns such as “Nursing Now” are valuable contributors to strengthening the role of nursing in healthcare teams to achieve better employment and working conditions for nurses [12].

Unionization in the health sector varies between regions, welfare state regimes and health workers groups, but regions have a similar trend of higher unionization in the public sector compared to the private one. For example, in Europe, the coverage of collective bargaining is notably higher in the public compared to the private sector. Collective agreement coverage in the private sector is considerably low and is even lower for nurses, for example, in the case of Poland, the coverage is only 5% [80].

Furthermore, while nursing students were employed as actual workers during the COVID-19 crisis, they were not always covered by the existing labour laws and the coverage of collective bargaining in this sector of workers is unknown. For example, a press release from the Irish Nurses’ and Midwives’ Organisation (INMO) points out that students “do not have the protections provided to employee” [42]. In Mexico, students have claimed that they are not given adequate PPE and hospitals told them that if they leave work, their scholarship will be withdrawn [81]. However, in England, the unions reached an agreement with the Nursing and Midwifery Council (NMC) and chief nursing officers across the UK that enables more experienced students to work in the NHS, receive remuneration and that this work counts for their learning [82]. Less has been explored about the role of universities in allowing, enabling and even possibly encouraging their students to enter high-risk health environments [83].

### Policies

The International Labour Organization (ILO) Nursing Personnel Convention, 1977 (No. 149) and the accompanying Recommendation (No. 157) set standards for fair employment conditions for nursing personnel. Yet, to date only 41 countries have ratified the Convention. Also, just 20 countries out of 194 Member States of the WHO have reported measures in place to prevent attacks on health workers [12].

During COVID-19, countries’ general policies do not address nursing work and needs. For example, the current COVID-19 guidance from Public Health England states that a fluid-resistant surgical face mask is sufficient for non-aerosol-generating procedures [84]. The administration of specific therapies, like Entonox, is not classified as an aerosol-generating procedure; however, a midwife can spend up to 11 hours in an unventilated room with an asymptomatic woman wearing Entonox with no protection other than a surgical mask. Inadequate PPE has been shown to be a source of infection among healthcare workers [8].

In addition, the declaration of COVID-19 as an occupational disease has been uneven and several countries have not yet developed this policy [85].

### Government, economy, and political priorities

There is evidence of numerous links between the characteristics of welfare state regimes and the regulation of nurse and nursing professionalization, suggesting that the political context has to be acknowledged and addressed to significantly influence nursing employment and working conditions and health policy [86, 87]. In addition, the presence of a Government Chief Nursing Officer (GCNO) position and the existence of a nursing leadership programme to effectively take action in government actions, are associated with a stronger regulation of employment and working conditions for nurses and regulation of nursing education [12]. However, not all countries have a GCNO, and the rhetoric of the dominant groups (i.e. medical doctors) has traditionally been overrepresented in the decision-making compared to nursing [88]. It is illustrated again with the COVID taskforce of many countries where the involvement of nurses has been negligible or null in coordinating and supervising the governments’ efforts to monitor, prevent, contain, and mitigate the spread of COVID-19. This is for example the case of India [89].

Regarding the economic situation, the 2008 economic crisis led to neoliberal austerity measures imposed in many countries that significantly curtail government spending. One of the measures implemented set caps on employment in the public sector which had a significant impact in the precarization of nurses [90]. For example, in Croatia it resulted in overburdening employed nurses; the inability of newly graduated professional nurses to access employment and, therefore, an increase in migration and a deepening of the serious shortage of nursing personnel [91, 92]. Other effects of the economy in the current nursing workforce can be found in Mexico, where prioritization in hiring nurses’ aides instead of professional nurses started as a response to the economic development plans of the 1970s that resulted in policies set by Mexico state [93]. Several countries in South Asia also followed a similar trajectory [94]. In addition to the direct effect of the austerity measures, a majority of nurses women were also severely affected by shrinking social protection floors with an impact on childcare and elderly care.

The global dynamics in the economy also plays a role, more visible during the pandemic. For example, the country’s purchasing capacity, the availability of PPE or vaccine production capacities, and the international dispute over scarce health workers.

## Discussion and limitations

Nurses’ work is essential in the health system as it has been proved once again during the COVID-19 pandemic. Yet, the pandemic has also exposed historic vulnerabilities faced by many nurses’ workers worldwide which resulted in an unacceptable number of infections and deaths among nurses. The neoliberal austerity measures promote precarization and informalization of nursing work and worsened the vulnerability to health-damaging factors, with many countries still to ratify the ILO Nursing Personnel Convention, 1977 (No. 149) on fair employment conditions. However, the current body of evidence lacks a detailed understanding of the pathways and mechanisms on how precariousness might affect nurses’ health. This might be linked to the current limited capacity of health information systems, and the inability to collect, analyse and monitor precarious employment and the impact in terms of health, wellbeing, and equity.

Collective bargaining through participation in unions and networks has proven to be effective in demanding for fairer employment and working conditions, but such collective organizing and legal rights are still insufficient in many countries. In line with this, COVID-19 pandemic has sparked new solidarity actions by nurses to bring more attention to nurses’ concerns, with calls for post-pandemic international and nationally enforceable standards. Some examples of successful actions to increase the number of nurse-centred and nurse-safe spaces to raise concerns and thus improve nursing working conditions during COVID crisis are reported from countries such as Brazil [95], India [96, 97], or Ireland [98].

The adapted theoretical framework of employment, work and health provides general guidance and helps in understanding the complex causal relations of employment, work and nurses’ health to guide policies and interventions to achieve greater equity. However, it needs to be tailored to the specific historical processes of each country, region or area, and social dynamics of different labour markets. An example is the wage hierarchies and the regulation of nurse and nursing professionalization [99]. In addition, this conceptual framework must also be considered with a dynamic perspective of the life cycle.

This study acknowledges that the search strategy used for the identification of studies might lead to exclusion of few relevant studies, although the searches performed have been extensive and it has been complemented with the lived experiences of bedside nurses from countries across Asia, Europe, and Latin America. Also, the study was done entirely virtually, and the team was not able to retrieve information from physical archives.

Box 1 describes the main recommendations based on the findings of the study. General recommendations combining policies at different entry points (power relations, employment, working conditions and ill-health workers) need to be specified and contextualized for each territory, condition, and population. Also, international institutions, governments and political parties, unions, and civil society associations favouring fair employment relations are key actors in implementing effective policies leading to the reduction of employment-related health inequalities.

Box 1. Recommendations Changes in power relations in nursing which can occur between the main political and economic actors in a society:

- Public health policies embedded in broader social and economic development planning and public funding should be developed;
- Structural drivers of inequality that push most vulnerable nurses, namely migrant, ethnically diverse, younger and older women, into more precarious and exploitative work should be recognized and act upon by them;
- Public financing to support gender equity and the needs of nurses' workers should be directed to increase access to the social protection mechanisms adequately funded and fully operational, notably, kindergartens, homes for the elderly, public transport and public housing;
- Further investment in public health systems should include the lift of existing caps on employment in the public sector, prioritization of standard employment of nurses in the public health systems and ensure nurses are paid adequately;
- National GCNO and nursing leadership programmes should be developed to promote stronger regulation of nursing education and employment and working conditions for nurses;
- The right of nurses to organize and join trade unions should be protected and workplace democracy should be recognized.

Modification of employment and working conditions to reduce vulnerability to health-damaging factors:

- Countries should ratify the ILO Nursing Personnel Convention, 1977 (No. 149) and act accordingly;
- Nursing education should be accessible, free-of-charge and of good quality;
- International mechanisms should be in place to regulate nurses’ migration, such as the WHO Global Code of Practice on the International Recruitment of Health Personnel;
- Migration policies that are not discriminatory or punitive and ensure that nurses can access public services adequately should be put in place;

Interventions to reduce the unequal consequences of ill-health and wellbeing:

- Universal access to health care including occupational health in primary health care should be provided to nurses, including those informalized;
- COVID-19 should be declared as an occupational disease;
- National capacity of health information systems and the international interlinkages should be strengthened to collect, analyse and monitor precarious employment and the impact in terms of nurses’ health, wellbeing, and equity.

## Conclusion

Nurses’ health is negatively affected by their employment and working conditions which in turn are determined by the power hierarchy and nursing employment relations. Current situation aggravated by the COVID-19 pandemic requires of international and national enforceable standards, along with economic and health policies designed to substantially improve employment and working conditions for nurses and work–life balance, reducing the burden of nurses’ “second shift” within their families. Future research should analyse the pathways and mechanisms on how precariousness might affect nurses’ health and monitor the progress (or not) towards nurses’ health equity over time and evaluate the effects of different interventions between and within countries.

## Data Availability

Not applicable.
